# Schistosomicidal, hepatoprotective and antioxidant activities of the methanolic fraction from *Clerodendrum umbellatum* Poir leaves aqueous extract in *Schistosoma mansoni* infection in mice

**DOI:** 10.1186/s12906-015-0788-z

**Published:** 2015-07-24

**Authors:** Hermine Boukeng Jatsa, Christian Mérimé Kenfack, Distele Nadège Simo, Nestor Gipwe Feussom, Emilienne Tienga Nkondo, Louis-Albert Tchuem Tchuente, Christelle Dongmo Tsague, Etienne Dongo, Pierre Kamtchouing

**Affiliations:** Laboratory of Animal Physiology, Department of Animal Biology and Physiology, Faculty of Science, University of Yaoundé I, P.O. Box 812, Yaoundé, Cameroon; Laboratory of Biology, Department of Animal Biology and Physiology, Faculty of Science, University of Yaoundé I, P.O. Box 812, Yaoundé, Cameroon; Centre for Schistosomiasis and Parasitology, P.O. Box 7244, Yaoundé, Cameroon; Laboratory of Phytochemistry, Department of Organic Chemistry, Faculty of Science, University of Yaoundé I, P.O. Box 812, Yaoundé, Cameroon

**Keywords:** *Clerodendrum umbellatum*, Liver function, Antioxidants, Oxidative stress, *Schistosoma mansoni*

## Abstract

**Background:**

The intensive use of Praziquantel for the treatment of schistosomiasis has raised concerns about the possible emergence of drug-resistant schistosomes. As drug treatment is an important feature of schistosome control programs, the search for alternative drugs is therefore a priority. The aim of this study was to assess the schistosomicidal, hepatoprotective and antioxidant activities of the methanolic fraction from *Clerodendrum umbellatum* Poir leaves aqueous extract.

**Methods:**

A phytochemical screening of the fraction of *C. umbellatum* was conducted. The fraction was administered orally and daily to *Schistosoma mansoni*-infected mice (BALB/c) from the 36th day post-infection for 28 days at 100, 200 and 400 mg/kg. Praziquantel (500 mg/kg) was used as reference drug. Non-infected and infected-untreated mice served as controls. All mice were sacrificed at 65th day post-infection. Body weight, liver/body and spleen/body weights, as well as worm burden, fecal egg count, liver and intestine egg load were determined. In the plasma, levels of total protein, transaminases (ALT, AST), alkaline phosphatase and total bilirubin were monitored to assess the possibility of liver damage. Malondialdehyde (MDA), catalase (CAT) and glutathione (GSH) levels were measured in the liver as biomarkers of the oxidative stress.

**Results:**

The phytochemical analysis of the fraction from *C. umbellatum* aqueous leaves extract revealed the presence of alkaloids, flavonoids, cardiac glycosides, phenols, saponins, tannins and terpenoids. The worm burden, fecal egg count and egg load in the liver and intestine of infected mice treated with the fraction were significantly (*p* < 0.001) fewer than in infected-untreated mice. Only the highest-fraction dose reduced the worm and egg burdens in a similar way as praziquantel. Hepatosplenomegaly induced by *S. mansoni* infection was reduced by the treatment. The liver function on infected mice was ameliorate after administration of the fraction by significant reduction of ALT activity (35.43 to 45.25 %) and increase of total protein level (44.79 to 70.03 %). The methanolic fraction of *C. umbellatum* prevents the elevated MDA level induced by the infection while significant increase in catalase activity (297.09 to 438.98 %) and glutathione level (58.23 to 95.88 %) were observed after treatment.

**Conclusions:**

This study disclosed the schistosomicidal, hepatoprotective and antioxidant activities of the methanolic fraction from *C. umbellatum* leaves aqueous. These fraction’s activities were similar to those of praziquantel. This fraction can be considered as a promising source for schistosomicidal agents.

## Background

Schistosomiasis is a chronic and debilitating disease affecting more than 200 million people in tropics and subtropics, with 97 % of them living in Africa [1]. The chronic and debilitating nature of the disease has resulted in heavy expenditure in public health and economic productivity in developing countries, and has prompted the initiation of large scale control programs [2]. *Schistosoma mansoni* infection is characterized by the embolization of eggs from the intestine to the liver through the portal system. Most pathology in schistosomiasis is then attributed to the host reaction to the eggs. The toxic egg material destroys the host tissue cells and the antigenic material stimulates the development of large inflammatory reactions around the egg. At the site of inflammation, oxidative stress occurs and lead to the generation of free radicals and the reduction of endogenous antioxidants [3, 4]. Presently, there is no available vaccine against schistosomiasis and the current treatment relies on praziquantel. However, praziquantel does not treat early infection or prevent reinfection [5]. In addition to, numerous evidences indicate the emergence of *Schistosoma mansoni* strains resistant to praziquantel [6, 7]. Therefore, there is growing consensus that novel antischistosomal drugs should be discovered and developed [8]. During *Schistosoma mansoni* infection, mice are good definitive host and their behavior is similar to that of humans. Thereby, the mouse is the animal of choice for *in vivo* antischistosomal drug screening [9]. In view to search complementary and/or alternative therapy for schistosomiasis, many studies on traditional medicinal plants have been conducted [10–15]. Previous ethnobotanical surveys revealed common use of plant materials in Africa for the treatment of intestinal helminthiasis. In Cameroon, the leaves of *Clerodendrum umbellatum* Poir (Labiateae) are among the common medicinal plants used by traditional healers to treat intestinal helminthiasis [16]. Our previous study has established the antischistosomal activity of *C. umbellatum* leaves aqueous extract [12]. The present study was carried out to evaluate the schistosomicidal, hepatoprotective and antioxidant activities of the methanolic fraction from *C. umbellatum* leaves aqueous extract on *Schistosoma mansoni* infected mice.

## Methods

### Plant material, extraction and fractionation

*Clerodendrum umbellatum* was collected in April 2012 at the locality of Mekok, near Sangmelima in the South region of Cameroon. The plant was identified at the National Herbarium of Yaoundé, Cameroon in comparison with the specimen 7405 SRF/Cam and conserved under the voucher N° 7405.

Leaves were dried in the shade, powdered and mixed with water (100 g/L) for 24 h of maceration at room temperature. The solution was filtered, frozen and then lyophilized to obtain the aqueous extract, with a recovery rate of 17 %. The aqueous extract (120 g) was fractionated by liquid–liquid partition with solvents of increasing polarity: *n*-hexane, ethyl acetate and methanol. Solvents were removed in a rotatory evaporator, at maximum temperature of 40 °C. This process allowed obtaining 31.57 g of a methanolic fraction.

### Phytochemical screening

The methanolic fraction from *C. umbellatum* leaves aqueous extract was subjected to qualitative chemical tests to identify phytochemical constituents in the fraction. The screening of alkaloids, anthraquinones and cardiac glycosides was performed by the Mayer’s test, the Bornträger’s test and the Keller-Killiani’s test respectively. Ferric chloride test (FeCl_3_) was used for the identification of phenols and tannins, Fehling’s test for reducing sugars, foam test for saponins and Liebermann-Burchard’s test for steroids and triterpenes. The presence of flavonoids, lipids and terpenoids was performed using the ammonia test, the grease spot test and the Salkowski’s test respectively [17].

### Animals and infection

Eight weeks old BALB/c mice from the animal house of the “Centre for Schistosomiasis and Parasitology” of Yaoundé–Cameroon, weighing 20–25 g were used in this study. They were housed in polypropylene cages in a bioterium under natural 12 h light/12 h dark cycles, temperature maintained between 22 and 25 °C and humidity between 60 and 70 %. Mice were fed with rodents’ diet (flour of corn, wheat, dried fish and roasted soybeans, cotton oil, mineral and vitamins mixture) and water *ad libitum*. We decided to work with BALB/c mice because they are susceptible to *S. mansoni* infection [12]. Mice were individually infected with 50 cercariae of *S. mansoni* (Cameroonian strain) using the method of tail and legs immersion. In brief, mice were first put in contact with water to stimulate urination and defecation. After that, the tail and hind legs of each mouse were immersed for 1 h in water containing 50 cercariae. Cercariae were released from *Biomphalaria pfeifferi* snails, collected from the river Afeme (Yaoundé, Cameroon) and maintained in the laboratory under standardized conditions.

### Ethical considerations

All procedures in this study followed the ARRIVE guidelines (Animal Research: Reporting *In Vivo* Experiments) and were approved by the Animal Ethical Committee of the Laboratory of Animal Physiology of the Faculty of Sciences, University of Yaoundé I–Cameroon.

### Experimental design

A total of 35 mice were used: 5 healthy mice (NIC group) and 30 *S. mansoni*-infected mice randomly divided into 5 groups of 6 mice each. Treatment started at the 36th day post-infection and the extract or praziquantel dissolved in distilled water was given by the oral route. Non-infected mice (NIC group) and infected-untreated mice (IC group) received distilled water during 28 consecutive days. The positive control group (PZQ group) received praziquantel at the dose of 500 mg/kg (100 mg/kg/day for 5 days, followed by distilled water for 23 days). Others infected mice were treated with the methanolic fraction from *C. umbellatum* leaves aqueous extract respectively at the doses of 100 (CuF100 group), 200 (CuF200 group) and 400 mg/kg (CuF400 group) during 28 consecutive days. This period of treatment was chosen according to the prescription of traditional healers and the choice of doses was done on the basis of our previous findings [12]. All animals were sacrificed on the 65th day post-infection.

### Measurement of body and organs weights and collection of specimens

During the course of the experiment, each mice was weighed once a week in order to assess body weight variation between pre-infection and post-infection at 65th day. The percentage of weight gain was calculated as follows: P = [(W_e_ − W_i_)/W_e_] × 100; where P is the percentage of weight gain, W_e_ is the body weight at post-infection and W_i_ is the body weight at pre-infection.

Fecal samples were collected from each mouse before sacrifice and processed for eggs counting. Blood from each animal was collected in the retro-orbital sinus and after centrifugation at 3500 rpm for 15 min, plasma were immediately stored at −70 °C until biochemical analyses were performed. Mice were sacrificed by cervical dislocation and after worm recovery, liver and spleen were removed from each mouse, weighed and their relative weights (g of organ/100 g of body weight) were calculated. The left lobe of the liver and the entire intestine were used to assess egg load while the right lobe of the liver was used for the assessment of hepatic oxidative stress.

### Worm recovery

Immediately after sacrifice on the 65th day post-infection, mice were perfused in order to recover worms from mesenteric and hepatic veins [18]. The percentage of reduction in worm number was calculated using the method of Tendler et al. [19] as follows: P = [(C − V)/C] × 100; where P is the percentage of reduction, C is the mean number of worms recovered from infected-untreated mice and V is the mean number of worms recovered from infected-treated mice.

### Eggs count in feces, intestine and liver

Feces were weighed, homogenized in 10 % buffered formaldehyde, and stored at 4 °C. Two aliquots of 100 μL each were counted on light microscope. The liver and the intestine which was previously cleaned up and weighed, were digested separately in 4 % KOH solution at 37 °C for 6 h. After digestion, tissue suspensions were centrifuged at 1500 rpm for 5 min and supernatants removed. After three cycles of washing and centrifugation, the number of eggs was determined in two aliquots of 100 μL each using light microscope. Results were expressed in terms of mean number of eggs per gram of feces or per gram of tissue for intestine and liver [20].

### Tissue homogenate

The right lobe of the liver was homogenized in Tris–HCl 50 mM buffer. Homogenates were centrifuged at 3500 rpm for 25 min at 4 °C and supernatants were stored at −70 °C for the determination of some oxidative stress biomarkers.

### Liver function tests

In view of evaluating the impact of the treatment with the methanolic fraction of *C. umbellatum* on the liver function of *S. mansoni*-infected mice, some parameters were measured in the plasma on the 65th day post-infection.

Plasma level of total proteins was determined using the method of Biuret described by Gornall et al. [21]. Biuret reagent was added to 10 μL of plasma from different mice groups and the developed purple color was measured after 5 min at 540 nm against a blank. The amount of protein was calculated from a standard curve using serial concentration of bovine serum albumin (0.25–1.5 mg/mL).

Alanine aminotransferase (ALT) and aspartate aminotransferase (AST) are known to be indicators of liver injuries. Their activities were determined by the Reitman and Frankel method [22] using Bioclin kits (Belo Horizonte, Brasil). ALT and AST were measured by monitoring the concentration of pyruvate hydrazone or oxaloacetate hydrazone formed with 2,4-dinitrophenylhydrazine, the color of which is read at 505 nm.

Alkaline phosphatase was estimated by the method of Tietz et al. [23] using Inmesco kit (Wied, Germany). A mixture of p-nitrophenylphosphate (16 mmol/L), 2-amino-2-methyl-1-propanol (0.9 mol/L), magnesium sulfate (1.6 mmol/L), zinc sulfate (0.6 mmol/L) and HEDTA (2 mmol/L) was used as the working solution. The decrease in absorbance was measured at 410 nm at 1 min intervals for 3 min.

Total bilirubin was assayed according to the protocol described by the Inmesco kit (Wied, Germany). In brief, 100 μL of sulfanilic acid + hydrochloric acid was added to a tube, followed by 20 μL of sodium nitrite, 400 μL of caffeine + sodium benzoate and 100 μL of the plasma. The contents were homogenized and allowed to stand for 5 min. The red color developed after the reaction of bilirubin with sulfanilic acid was read at 546 nm against a blank.

### Lipid peroxidation, glutathione status and catalase activity determination

Using murine model of schistosomiasis, it has been demonstrated that *S. mansoni* induces hepatic oxidative stress due to the production of reactive oxygen species and the reduction of the antioxidant defense processes of the organs [3, 4]. The impact of the treatment of *S. mansoni*-infected mice with the methanolic fraction of *C. umbellatum* on their hepatic oxidative stress was then assessed on the 65th day post-infection.

Lipid peroxidation was estimated by determining malondialdehyde (MDA). 250 μL of 20 % trichloroacetic acid and 500 μL of 0.67 % thiobarbituric acid were added to 500 μL of the supernatant,. The tubes were boiled in a water bath at 90 °C for 10 min. After cooling on ice-cold water, the mixture was centrifuged at 3000 rpm for 15 min. The optical density was measured at 530 nm against an appropriate blank [24]. The concentration of MDA was calculated using a molar extinction coefficient of 1.56 × 10^5^ mmol^−1^ cm^−1^ and results expressed as nmol/g of liver.

Reduced glutathione (GSH) was assayed following the method described by Ellman [25]. 1500 μL of Ellman reagent (5 mg of 2, 2-dithio-5, 5-nitrobenzoic acid + 250 mL phosphate buffer) was added to 100 μL of the supernatant,. The mixture was shaken and kept for 60 min. Optical density was measured at 412 nm against a blank. The concentration of GSH was calculated using a molar extinction coefficient of 13,600 mol^−1^ cm^−1^ and results expressed as μmol/g of liver.

The reaction for assaying catalase activity as described by Sinha [26], was initiated by adding 187.5 μL of phosphate buffer to 12.5 μL of the supernatant. The reaction started when 50 μL of H_2_O_2_ 50 mM was added to the mixture and stopped by adding 500 μL of dichromate-acetic acid mixture (50 mL of potassium dichromate 5 % dissolved in 150 mL of glacial acetic acid). Standards tubes were prepared using a solution of H_2_O_2_ 50 mM. The optical density was recorded at 570 nm. Catalase activity was calculated from the standard curve and expressed as mmol/min/g of liver.

### Statistical analysis

All data were expressed as mean ± standard error of mean (SEM). Statistical differences between controls and experimental groups were assessed by one-way analysis of variance (ANOVA) followed by Newman-Keuls multiple comparison test. *P* values less than 0.05 (*p* < 0.05) were considered to be significant. Analysis was performed using GraphPad Prism version 4.00 for Windows (GraphPad Software, San Diego, USA).

## Results and discussion

### Phytochemical analysis of the methanolic fraction from *Clerodendrum umbellatum* leaves aqueous extract

The qualitative phytochemical analysis of the methanolic fraction from *C. umbellatum* leaves aqueous extract revealed the presence of alkaloids, flavonoids, cardiac glycosides, phenols, saponins, tannins and terpenoids. Except phenols, those compounds have previously been found in *C. umbellatum* aqueous extract [12].

### Effect of the methanolic fraction from *Clerodendrum umbellatum* leaves aqueous extract on the body weight

Epidemiological studies have shown that *S. mansoni* infection is generally associated with stunting among children [27]. This is also noticed in *S. mansoni*-infected mice [15]. Over the course of the study, all groups showed weight gain. However, the weight gain of infected-untreated animals (IC) was significantly (*p* < 0.001) lower than the one of healthy mice (NIC). There were also significant differences between the weight gain of infected-treated animals with praziquantel or the methanolic fraction of *C.umbellatum* at 100, 200 and 400 mg/kg and that of infected-untreated animals (IC) (Table 1). Results from our study demonstrated that treatment with praziquantel or *C. umbellatum* improves on the growth of *S. mansoni*-infected animals. Similar findings were previously reported by Rizk et al. [15] with the essential oil of *Melaleuca armillaris* fresh leaves.

**Table 1:** Effect of the methanolic fraction from *Clerodendrum umbellatum* leaves aqueous extract on the body weight of mice at pre-infection and on the 65th day post-infection

Groups	Number of animals (*n*)	Body weight (g)		Weight gain (%)	*p*
		Pre-infection (1st day)	Post-infection (65th day)		
NIC	5	19.30 ± 0.83	24.23 ± 1.05	29.53 ± 2.04 (24.28–34.78)	
IC	6	23.51 ± 0.90	24.31 ± 0.80	3.97 ± 3.87 (−5.50–13.44)	<0.001
PZQ	6	21.22 ± 1.04	26.00 ± 0.98	22.89 ± 2.89 (15.47–30.31)	<0.001
CuF100	6	23.55 ± 0.55	28.14 ± 0.85	19.54 ± 2.56 (12.95–26.12)	<0.01
CuF200	6	21.04 ± 0.89	24.94 ± 1.03	18.60 ± 1.43 (14.93–22.26)	<0.001
CuF400	6	22.74 ± 0.51	27.07 ± 0.74	19.06 ± 1.96 (14.03–24.09)	<0.001

Data are expressed as mean ± SEM

Values in brackets represent the 95 % confidence intervals

ANOVA followed by Newman-Keuls multiple comparison test was used to compare on one hand, the infected-untreated mice (IC) to the non-infected mice (NIC), and secondly the infected-treated mice (PZQ, CuF100, CuF200, CuF400) to the infected-untreated mice (IC)

### Effect of the methanolic fraction from *Clerodendrum umbellatum* leaves aqueous extract on the liver and spleen weights

To assess the effect of the methanolic fraction of *C. umbellatum* on the hepatosplenomegaly induced by *S. mansoni* infection, relative weights of liver and spleen were evaluated. Results from this study showed that infected-untreated mice presented significant (*p* < 0.001) increase in both relative liver and spleen weights by 47.83 and 190 % respectively when compared to those of non-infected mice (Table 2). Many authors have reported that one manifestation of a high *S. mansoni* infection intensities in school-aged children and adults is hepatosplenomegaly [28, 29]. Enlargement of the liver and spleen is the consequence of the deposition of numerous schistosome eggs inside the tissues, which provoked chronic granulomatous inflammation [30]. Moreover, the underlying process causing hepatosplenomegaly also has a detrimental effect on the growth of children since the production of inflammatory cytokines adversely affects the production of the growth hormone IGF-1 by the liver [29]. This mechanism could therefore explain the growth retardation of infected-untreated mice. The liver and spleen weights of infected-treated animals with praziquantel or the methanolic fraction of *C. umbellatum* at 100, 200 and 400 mg/kg were significantly lower than those of infected-untreated animals (Table 2). These results indicate that the treatment with praziquantel or the fraction alleviates hepatosplenomegaly induced by *S.mansoni* infection. Previous study with the aqueous extract of *C. umbellatum* leaves have shown similar result [12].

**Table 2:** Effect of the methanolic fraction from *Clerodendrum umbellatum* leaves aqueous extract on the liver and spleen weights of mice on the 65th day post-infection

Groups	Number of animals (*n*)	Liver			Spleen		
		Weight (g/100 g)	*p*	% change	Weight (g/100 g)	*p*	% change
NIC	5	6.23 ± 0.34			0.50 ± 0.04		
IC	6	9.21 ± 0.37	<0.001	+47.83	1.45 ± 0.17	<0.001	+190.00
PZQ	6	6.57 ± 0.46	<0.001	−28.66	0.43 ± 0.08	<0.001	−70.34
CuF100	6	7.68 ± 0.28	<0.05	−16.61	0.77 ± 0.08	<0.001	−46.90
CuF200	6	7.90 ± 0.48	<0.05	−14.22	0.79 ± 0.03	<0.001	−45.52
CuF400	6	5.96 ± 0.47	<0.001	−35.29	0.66 ± 0.10	<0.001	−54.48

Data are expressed as mean ± SEM

ANOVA followed by Newman-Keuls multiple comparison test was used to compare on one hand, the infected-untreated mice (IC) to the non-infected mice (NIC), and secondly the infected-treated mice (PZQ, CuF100, CuF200, CuF400) to the infected-untreated mice (IC)

### Effect of the methanolic fraction from *Clerodendrum umbellatum* leaves aqueous extract on worm burden and egg load

**Fig. 1:**
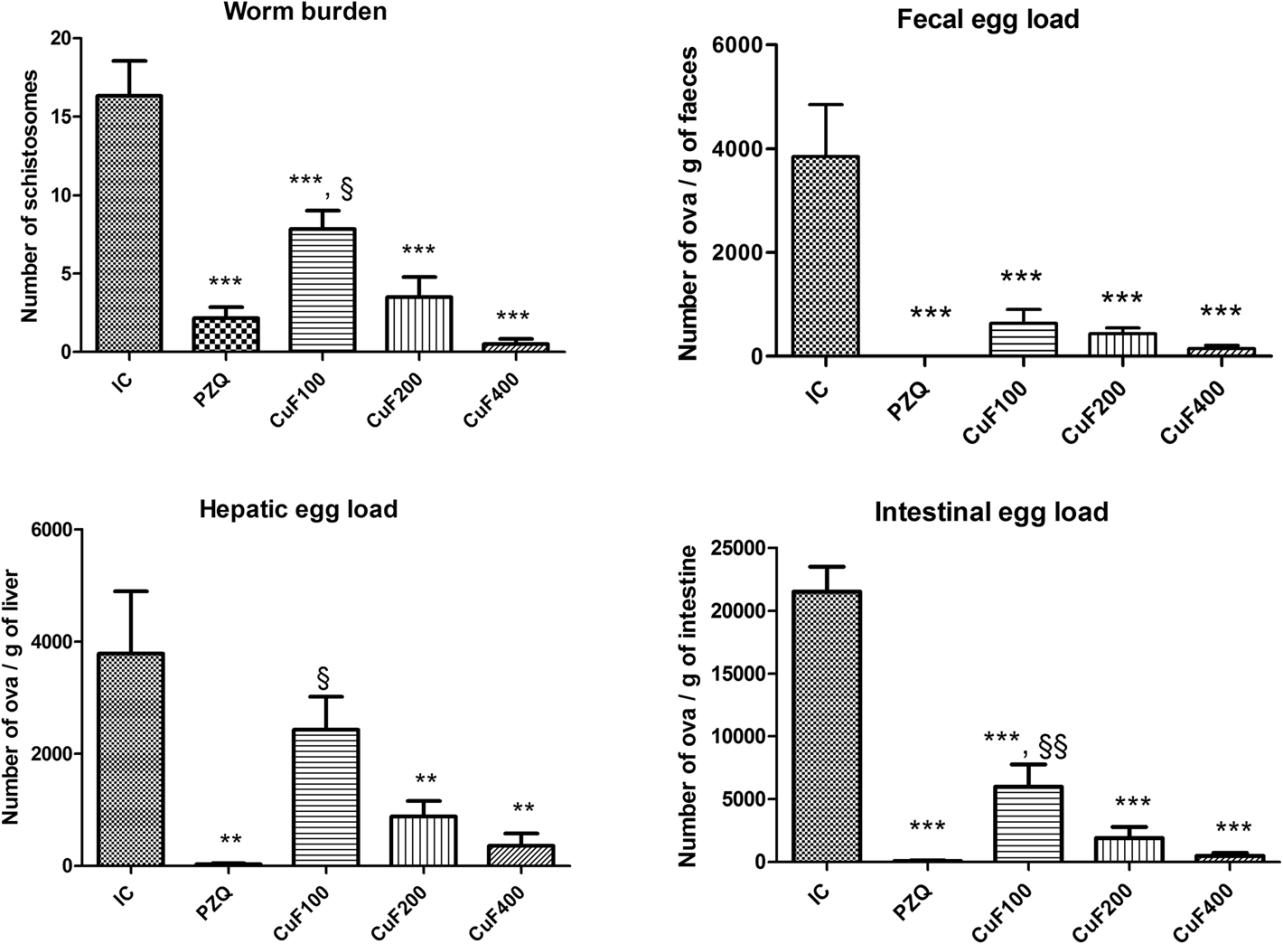
Effect of the methanolic fraction from *Clerodendrum umbellatum* leaves aqueous extract on the worm burden, fecal egg count and egg load in the liver and intestine of mice on the 65th day post-infection. Data are expressed as mean ± SEM; *n* = 5 (NIC) or *n* = 6 (IC, PZQ, CuF100, CuF200 and CuF400). *NIC* non-infected mice, *IC* infected-untreated mice, *PZQ* infected mice treated with praziquantel; CuF100, CuF200 and CuF400, infected mice treated with the methanolic fraction from *C. umbellatum* leaves aqueous extract at 100, 200 and 400 mg/kg respectively. ANOVA followed by Newman-Keuls multiple comparison test: ^**^, ^***^: statistically different from IC group at *p* < 0.01 and *p* < 0.001 respectively. ^§^, ^§§^: statistically different from PZQ group at *p* < 0.05 and *p* < 0.01 respectively

Treatment of infected mice with the methanolic fraction of *C. umbellatum* was followed by reduction of worm burden by 52.05, 78.57 and 96.94 % at doses of 100, 200 and 400 mg/kg respectively. The mortality rate of schistosomes after praziquantel treatment was 86.71 % (Fig. 1). Significant reduction of egg load in the feces, the liver and the intestine were recorded after treatment of infected animals with praziquantel or *C. umbellatum* at all doses. When compared to infected-untreated animals, mice treated with the methanolic fraction from *C. umbellatum* leaves aqueous extract at 400 mg/kg showed reduction of egg load by 96.34 % in the feces, 90.53 % in the liver and 97.72 % in the intestine. In the PZQ group, no egg was seen in the feces and reduction rates of the egg output were 99.34 % in the liver and 99.67 % in the intestine (Fig. 1). The reduction of worms is generally correlated to the reduction of ova in the feces and tissues, since there is a positive linear relationship between the egg output and the worm burden [13]. By reducing the number of *S. mansoni* ova in the liver and intestine, *C. umbellatum* may contribute to the improvement of hepatosplenomegaly. Reduction of worm and egg load was also found by many authors after treatment of *S. mansoni*-infected animals with medicinal plants extracts [10, 11, 13, 14]. Among pharmacological activities of chemical compounds, alkaloids and tannins are known to possess anthelmintic activity. Schistosomicidal activity of alkaloids has been proved by Miranda et al. [14] while reduction of worm motility and depression of egg output have been correlated to the presence of tannins in plants [31]. Alkaloids present in the methanolic fraction of *C. umbellatum* could act as praziquantel by inducing schistosome mortality through general paralysis or proteolysis [32].

### Effect of the methanolic fraction from *Clerodendrum umbellatum* leaves aqueous extract on the liver function

Comparatively to non-infected mice, infected-untreated mice showed a significant increase (*p* < 0.001) of ALT activity by 180.18 % and a significant decrease (*p* < 0.001) of total proteins level by 28.44 %. AST activity also increased by 39.55 % in infected-untreated mice, but this was not statistically significant (Table 3). Previous studies have revealed increase of serum ALT and AST activities [11] as well as the reduction of total proteins concentration in the liver following *S. mansoni* infection [10, 15]. When the hepatocellular membrane is damaged, enzymes normally present in the cytosol are released into the blood stream. The elevated transaminases (ALT and AST) activities in infected-untreated animals could be related to such effect. Rizk et al. [33] have reported that proteins concentration in *S. mansoni*-infected mice begin to decline at six weeks post-infection as a result of a reduction of protein anabolism and increase of protein catabolism. *S. mansoni*-infected mice also showed decrease of ALP activity and increase of bilirubin concentration by 57.73 and 19.70 % respectively, despite the fact that this was not statistically significant (Table 3). Thapa and Walia [34] have reported that reduced ALP activity was a result of severe anemia which is one of the main features of schistosomiasis mansoni. Results from this study demonstrated that granulomatous response initiated by the presence of eggs in the liver during *S. mansoni* infection impairs the liver function and it secretory activity. Administering of either praziquantel or the methanolic fraction from *C. umbellatum* leaves aqueous extract at 100, 200 and 400 mg/kg to infected mice induced significant reduction (*p* < 0.001) of ALT activity and elevation of plasma total proteins concentration (*p* < 0.001), when compared to those of infected-untreated mice. Activity of ALP was also ameliorated by PZQ and *C. umbellatum* treatments (Table 3). The methanolic fraction from *C. umbellatum* leaves aqueous extract could then contribute to improve liver function by preserving hepatocytes integrity as well as repairing hepatic damages caused by the infection. Antiinflammatory compounds such as phenols, flavonoids and tannins present in the fraction may contribute to the regeneration of hepatocytes [35].

**Table 3:** Effect of the methanolic fraction from *Clerodendrum umbellatum* leaves aqueous extract on some parameters of the liver function of mice on the 65th day post-infection

Groups	NIC	IC	PZQ	CuF100	CuF200	CuF400
Number of animals (*n*)	5	6	6	6	6	6
ALT (U/mL)	266.28 ± 9.90	746.07 ± 59.86	391.66 ± 31.85	408.49 ± 36.27	420.52 ± 26.68	481.76 ± 47.09
*p*		<0.001	<0.001	<0.001	<0.001	<0.001
% change		+180.18	−47.50	−45.25	−43.63	−35.43
AST (U/m)	167.84 ± 10.55	234.23 ± 14.58	168.13 ± 13.41	234.52 ± 23.74	248.24 ± 20.11	261.75 ± 16.25
*p*		n.s	n.s	n.s	n.s	n.s
% change		+39.55	−28.22	+0.12	+5.98	+11.75
ALP (U/L)	19.02 ± 2.87	8.04 ± 2.88	20.68 ± 3.24	13.10 ± 2.90	18.38 ± 2.86	20.33 ± 4.07
*p*		n.s	n.s	n.s	n.s	n.s
% change		−57.73	+157.21	+62.93	+128.61	+152.86
Total proteins (mg/mL)	4.43 ± 0.22	3.17 ± 0.29	4.73 ± 0.15	4.88 ± 0.18	5.39 ± 0.18	4.59 ± 0.16
*p*		<0.001	<0.001	<0.001	<0.001	<0.001
% change		−28.44	+49.21	+53.94	+70.03	+44.79
Total bilirubin (μmol/L)	19.34 ± 1.71	23.15 ± 1.80	15.32 ± 1.76	16.87 ± 3.78	20.84 ± 1.50	25.59 ± 3.88
*p*		n.s	n.s	n.s	n.s	n.s
% change		+19.70	−33.82	−27.13	−9.98	+10.54

Data are expressed as mean ± SEM

ANOVA followed by Newman-Keuls multiple comparison test was used to compare on one hand, the infected-untreated mice (IC) to the non-infected mice (NIC), and secondly the infected-treated mice (PZQ, CuF100, CuF200, CuF400) to the infected-untreated mice (IC). *n.s* non-significant

*ALT* alanine aminotransferase, *AST* aspartate aminotransferase, *ALP* alkaline phosphatase

### Effect of the methanolic fraction from *Clerodendrum umbellatum* leaves aqueous extract on some markers of oxidative stress

**Fig. 2:**
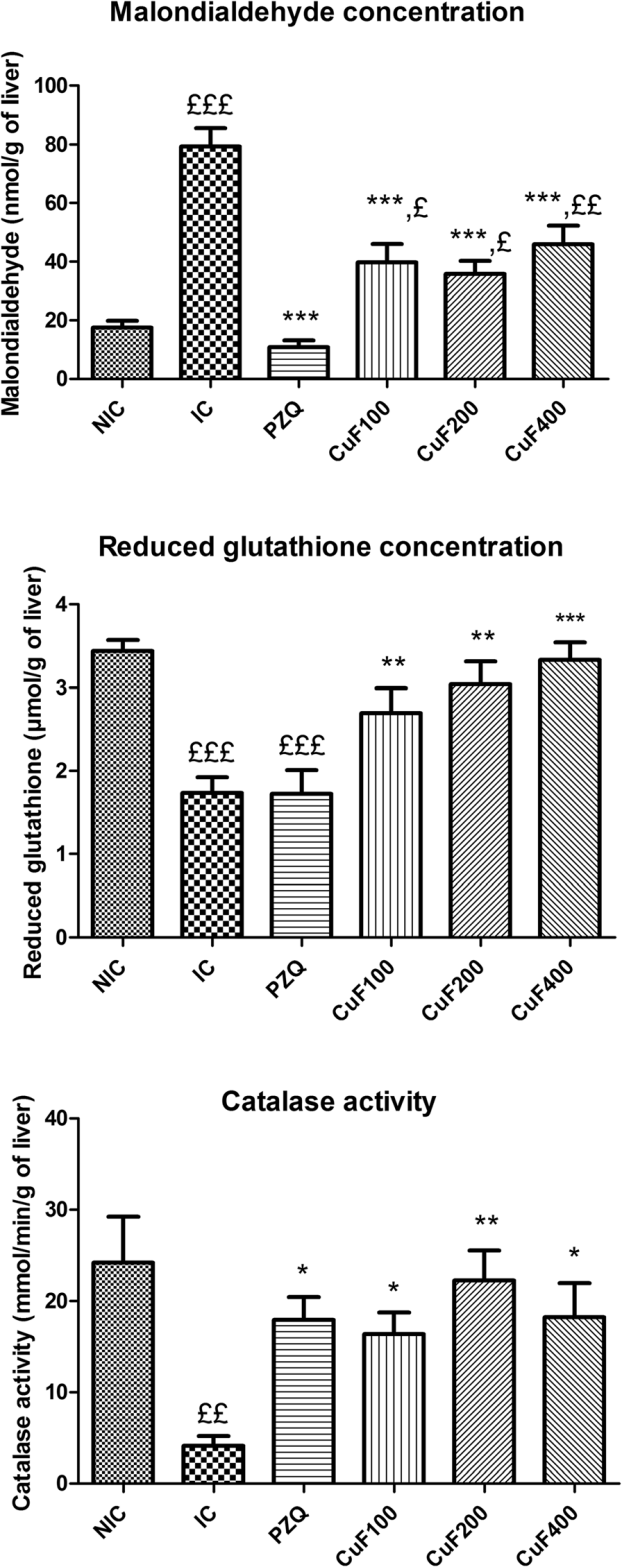
Effect of the methanolic fraction from *Clerodendrum umbellatum* leaves aqueous extract on some biomarkers of the oxidative stress on the 65th day post-infection. Data are expressed as mean ± SEM; *n* = 5 (NIC) or *n* = 6 (IC, PZQ, CuF100, CuF200 and CuF400). *NIC* non-infected mice, *IC* infected-untreated mice, *PZQ* infected mice treated with praziquantel; CuF100, CuF200 and CuF400: infected mice treated with the methanolic fraction from *C. umbellatum* leaves aqueous extract at 100, 200 and 400 mg/kg respectively. ANOVA followed by Newman-Keuls multiple comparison test: ^£^, ^££^, ^£££^: statistically different from NIC group at *p* < 0.05, *p* < 0.01 and *p* < 0.001 respectively. ^*^, ^**^, ^***^: statistically different from IC group at *p* < 0.05, *p* < 0.01 and *p* < 0.001 respectively

As shown of Fig. 2, malondialdehyde concentration was significantly (*p* < 0.001) increased by 352.37 % (79.30 ± 6.30 vs 17.53 ± 2.30 nmol/g of liver) in infected-untreated mice comparatively to non-infected mice. There were also significant reductions of glutathione concentration (*p* < 0.001) by 49.71 % (1.73 ± 0.19 vs 3.44 ± 0.13 μmol/g of liver) and catalase activity (*p* < 0.01) by 82.92 % (4.13 ± 1.07 vs 24.18 ± 5.03 mmol/min/g of liver). These results are in agreement with previous data on humans with schistosomal hepatic fibrosis and murine models of schistosomiasis [11, 15, 33, 36–38]. Increased malondialdehyde concentration could be the consequence of the release of significant amount of superoxide radicals by macrophages of hepatic granulomas in *S. mansoni* infection [36]. Catalase and reduced glutathione are endogenous antioxidants of cellular antioxidant defence. Depletion of their levels indicate an increase in free radicals level and thereby increase of cellular damage [11, 36]. Comparatively to infected-untreated mice, infected mice who received either praziquantel or the methanolic fraction from *C. umbellatum* leaves aqueous extract at 100, 200 and 400 mg/kg showed significant (*p* < 0.001) decrease of hepatic malondialdehyde level by 86.42, 50, 54.93 and 42.05 % respectively. This fraction also suppressed, in a dose-dependent manner, the depletion of the reduced glutathione in the liver of infected mice. When compared to that of infected-untreated mice, the glutathione level of infected mice treated with the fraction at 100, 200 and 400 mg/kg increased by 58.23 % (*p* < 0.01), 78.82 % (*p* < 0.01) and 95.88 % (*p* < 0.001) respectively. Praziquantel failed to prevent the depletion of the reduced glutathione in the liver of infected mice, but increased the catalase activity by 334.38 % (*p* < 0.05) as compared to that of infected-untreated mice. Comparatively to infected-untreated mice, infected mice treated with *C. umbellatum* also exhibited significant elevation of catalase activity by 297.09, 438.98 and 341.16 % at 100, 200 and 400 mg/kg respectively. These findings clearly indicated that the methanolic fraction from *C. umbellatum* leaves aqueous extract prevented the modification of malondialdehyde, reduced glutathione and catalase levels induced by *S. mansoni* infection, suggesting its antioxidant properties. This fraction probably protects the hepatic tissue against oxidative damage by scavenging reactive hydroxyl and peroxyl radicals [11]. Phenolic compounds are the main agents that can donate hydrogen to free radicals and thus break the chain reaction of lipid oxidation at the first initiation step. This high potential of phenolic compounds to scavenge radicals may be explained by their phenolic hydroxyl groups [39]. Based on the results of our phytochemical analysis, flavonoids, phenols and tannins could be responsible for the significant antioxidant activity of the methanolic fraction from *Clerodendrum umbellatum* leaves aqueous extract.

## Conclusions

This study showed that the methanolic fraction from *Clerodendrum umbellatum* leaves aqueous extract exhibits schistosomicidal, hepatoprotective and antioxidant activities in *Schistosoma mansoni* infection. These activities are probably related to bioactive compounds present in the fraction. Schistosomicidal activity similar to that of praziquantel was displayed by the highest dose of the fraction. Therefore, this fraction could be used as a starting point for the development of phytomedicines and/or source of new molecules against schistosomiasis.
